# Fracture strength of roots restored with relined and milled glass fiber posts

**DOI:** 10.3389/fdmed.2026.1711272

**Published:** 2026-02-26

**Authors:** Pedro Henrique Soares Aguiar, Kusai Baroudi, Tarun Walia, Vivek Padmanabhan, Fillipe Mendes Silva, Ellen Christine Rodrigues de Abreu, Milton Edson Miranda, Rafael Pino Vitti, William Cunha Brandt

**Affiliations:** 1 Faculdade São Leopoldo Mandic, Instituto de Pesquisas São Leopoldo Mandic, Campinas, São Paulo, Brazil; 2 Department of Clinical Sciences, College of Dentistry, Ajman University, Ajman, United Arab Emirates; 3 Centre of Medical and Bio-allied Health Sciences Research, Ajman, United Arab Emirates; 4 Postgraduate Program in Health Sciences, School of Dentistry, University of Taubate, Taubate, São Paulo, Brazil; 5 Department of Pediatric and Preventive Dentistry, RAK College of Dental Sciences, RAK Medical and Health Sciences University, Ras Al Khaimah, United Arab Emirates; 6 Faculdades Unidas do Norte de Minas – Funorte, Minas Gerais, Brazil; 7 Department of Implantology, School of Dentistry, University Santo Amaro, São Paulo, Brazil

**Keywords:** CAD/CAM, fracture strength, intraradicular post, milled glass fiber post, relined glass fiber post

## Abstract

**Objectives:**

Computer-aided design/computer-aided manufacturing (CAD/CAM)-milled glass fiber posts offer notable advantages. However, their performance compared to that of prefabricated, composite-reinforced glass fiber posts is controversial. This study aimed to evaluate the fracture strength and fracture patterns of canine roots restored with relined glass fiber posts and CAD/CAM-milled glass fiber posts.

**Materials and methods:**

In total, 20 decoronated canine roots underwent endodontic treatment (*n* = 10). In the relined group (control), prefabricated glass fiber posts (Reforpost Angelus #1, Angelus, Brazil) were relined with a resin composite (Z350, 3M ESPE, USA). In the milled group, the root canal was modeled using a post (Pinjet, Angelus) and red acrylic resin (Pattern Bright Kit, Kota Ind., Brazil). The acrylic resin patterns were scanned and milled from a glass fiber block (Fiber CAD Post & Core, Angelus). A dual-cure self-adhesive resin luting agent (RelyX™ U200, 3M ESPE) was used for post cementation in both groups. The specimens were subjected to compressive loading, applied at a 45° angle to the long axis of the tooth using a universal testing machine with 200 kgf of compressive force at a crosshead speed of 1 mm/min, until failure. The failure mode was classified as catastrophic (fracture extended to the middle and/or apical third) or repairable (glass fiber post and/or coronal core or limited to the cervical region). A statistical analysis was performed using an independent samples *t*-test (*α *= 0.05).

**Results:**

A statistically significant difference was observed in the mean fracture strength, with higher values for the milled group (*p* = 0.004). Furthermore, the milled group had fewer catastrophic failures.

**Conclusions:**

The CAD/CAM-milled glass fiber posts exhibited greater fracture strength and a reduced incidence of catastrophic fractures.

## Introduction

Endodontically treated teeth often suffer from substantial coronal structure loss, which frequently necessitates the use of intraradicular posts to restore both function and aesthetics. The post is used to retain the core buildup and provide essential support for the restoration (1–3). Various types of posts have been introduced over the years, progressing from traditional metal alloys to advanced non-metallic options. While metallic posts were once commonly employed, they have exhibited notable limitations, such as a high modulus of elasticity. This characteristic can result in stress concentration, potentially causing catastrophic fractures in the remaining tooth structure (2, 4).

Glass fiber posts have emerged as the preferred material in contemporary dental practice due to their superior aesthetic qualities and biomechanical compatibility with dentin, presenting a modulus of elasticity similar to that of the natural tooth structure (5). This attribute helps minimize stress concentrations, thus reducing the risk of irreparable root fractures. In addition, the use of resin-based luting agents has further improved the long-term survival rates of teeth restored with glass fiber posts (6).

Both natural and restored teeth are subject to stress under masticatory forces. Glass fiber posts contribute to a more even distribution of these occlusal forces along the root, thereby reducing the risk of root fractures. Even when fractures do occur, glass fiber posts generally help to preserve root integrity, making subsequent repairs feasible (7, 8). Despite these benefits, prefabricated glass fiber posts have limitations due to the need for customization. The anatomy of the root canal can vary significantly between teeth, requiring clinicians to adapt the post with a resin composite to ensure an ideal fit (3). While effective, this approach introduces additional adhesive interfaces, potentially increasing the risk of dimensional inaccuracies. In addition, the diameter and height of the post play a critical role in the fracture resistance of the restored tooth, closely linked to the unique morphology of each root canal (9).

Advances in digital dentistry, particularly with the advent of computer-aided design (CAD) and computer-aided manufacturing (CAM), have introduced a novel approach to fabricating customized glass fiber posts. Using CAD/CAM technology, posts can be precisely milled from fiberglass blocks, creating a single-unit design that eliminates adhesive interfaces and optimizes the fit within the root canal (10). These milled posts exhibit superior biomechanical properties and reduce the required thickness of the resin-based luting agent during cementation, thereby minimizing the potential for polymerization stress (11).

Although CAD/CAM-milled glass fiber posts offer notable advantages, such as reducing adhesive failures and enhancing adaptation (10), few studies have directly compared their performance to that of prefabricated, composite-reinforced glass fiber posts. This gap in the literature highlights the need to assess the fracture strength and failure modes of these two post types to better understand their respective effectiveness and limitations. In particular, the presence of adhesive interfaces in prefabricated posts, as opposed to the monolithic structure of milled posts, may influence both fracture behavior and potential for repair.

This study aims to evaluate the fracture strength and failure modes of prefabricated glass fiber posts relined with a resin composite and CAD/CAM-milled glass fiber posts. The null hypothesis is that there will be no difference in fracture strength between the two types of posts.

## Materials and methods

### Experimental design

The sample size adopted in this study (*n* = 10) was based on previous *in vitro* investigations evaluating the fracture strength of endodontically treated teeth restored with intraradicular posts using similar experimental designs and outcome measures. Studies with comparable methodologies commonly report sample sizes ranging from eight to 12 specimens per group and demonstrate large effect sizes for fracture resistance outcomes. Based on these prior findings, a sample size of 10 specimens per group was considered sufficient to detect clinically and mechanically relevant differences under controlled laboratory conditions.

In addition to hypothesis testing, the magnitude of the difference between groups was assessed by calculating the effect size (Cohen's d) with corresponding 95% confidence intervals, allowing evaluation of the clinical and mechanical relevance of the observed differences independent of *post hoc* power considerations. Previous *in vitro* studies comparing different fiber post systems have consistently reported large differences in fracture resistance, supporting the expectation of large effect sizes in similar experimental designs (7, 11).

Root specimens from canine teeth rehabilitated with intraradicular posts were used. The study factors included intraradicular posts at the following two levels: glass fiber posts relined with resin composite (parallel posts with 80% glass fiber reinforced with a central metal filament and 20% epoxy resin) and CAD/CAM-milled glass fiber posts (80% unidirectional glass fiber and 20% epoxy resin). The response variables were the fracture strength of the roots rehabilitated with intraradicular posts and the failure mode analysis (categorized as repairable or catastrophic).

### Sample selection and preparation

This study involved the use of extracted human teeth and did not include any animal experimentation. In total, 20 human canine teeth were stored in 0.1% thymol solution under refrigeration after ethics committee approval (CAAE 66708123.2.0000.5374, College of Sao Leopoldo Mandic, Brazil). The teeth were selected according to the following criteria: no prior endodontic treatment, intact crowns, absence of restorations and cracks, a single canal, and similar shape and size. Radiographs were taken in the ortho-radial and mesio-distal positions to confirm the presence of a single canal, and a microscopic examination was performed to verify the absence of cracks.

After selection, each root was measured with a digital caliper (Digimess, Shiko Precision Gaging, China) and marked at a distance of 16 mm from the apical region. Following standardization markings on each root, a cross-sectional cut was made using a low-speed (350 rpm) diamond disc under water-cooling (Isomet 1000, Buehler, Lake Forest, IL, USA). The root specimens were then stored in polypropylene tubes (Eppendorf AG Brazil, São Paulo, SP, Brazil) containing 1 mL of distilled water. The tubes were kept in an incubator at 37°C for 72 h to ensure rehydration.

### Endodontic treatment

The dentin wall thickness was reduced (maintaining a minimum thickness of 2 mm) to standardize the diameter for intraradicular post preparation using 718 PM burs (KG Sorensen, São Paulo, SP, Brazil) under irrigation, with the bur inserted 8 mm into the canal. Root canal irrigation was performed using a 2.5% sodium hypochlorite solution, followed by its aspiration and re-insertion with #10K-file (Dentsply Maillefer, Baillagues, Switzerland) into the canal until its tip was visible at the apical foramen. The working length was determined for each sample by subtracting 1 mm from the total length traveled by the file during exploration. Thus, #15K-files (Dentsply Maillefer) were introduced to determine the anatomical diameter. The canals were further prepared using rotary instruments (Easy Produtos Odontológicos, Belo Horizonte, Brazil) operated with an electric motor (VDW Silver, VDW GmbH, Munich, Germany). The root canal was instrumented with the initial file up to size #40. After each file change, the canal was irrigated, aspirated, and flushed with 2 mL of 2.5% sodium hypochlorite.

Root canal preparation was finalized through instrumentation at the working length. The samples underwent syringe irrigation using Navi Tips (Ultradent Products, South Jordan, UT, USA) positioned 2 mm from the working length. Final irrigation consisted of 2 mL of 17% EDTA. The EDTA remained in the root canal for 5 min to ensure the effective and complete removal of the smear layer after extensive canal preparation and post space instrumentation. This extended application time was selected to standardize chelation under the enlarged canal conditions required for post placement. After EDTA application, the canals were thoroughly irrigated with distilled water to minimize the risk of excessive dentin demineralization. All the specimens were subjected to the same irrigation protocol to maintain full experimental standardization. Then, the canal was irrigated with 10 mL of distilled water and subsequently dried with absorbent paper points (Dentsply Maillefer, Baillagues, Switzerland) corresponding to instrument size #40.

The root canal obturation was performed with AH Plus endodontic sealer (Dentsply Sirona, USA) using the lateral condensation technique, with a main gutta-percha cone and accessory cones (Dentsply Maillefer, Baillagues, Switzerland). The gutta-percha was sectioned using a heated Hollenback #3 carver (Golgran, Brazil). Residual endodontic cement was cleaned with a 70% alcohol-moistened sponge. The quality of obturation was assessed through radiographic imaging. The roots were stored in Eppendorf-type polypropylene tubes (Eppendorf Brasil Ltda, São Paulo, SP, Brazil) containing 1 mL of distilled water in an incubator at 37°C and 100% humidity.

### Preparation of the root canal, fabrication of glass fiber posts, and cementation

Following endodontic treatment, the gutta-percha was removed to create space for the glass fiber post. Peeso reamer sizes 2, 3, and 4 (Dentsply GmbH, Konstanz, Germany) were used for post space preparation, preserving 4 mm of the apical gutta-percha, resulting in standardized post lengths of 11 mm. The 20 teeth were randomly assigned to two groups (*n* = 10):

Relined (control): Roots restored with prefabricated glass fiber posts relined with a resin composite.

Milled: Roots restored with CAD/CAM-milled glass fiber posts.

In both groups, a periapical radiograph was taken after post space preparation to confirm the presence of 4 mm of the apical gutta-percha. The canal was then isolated with a water-soluble gel applied with a brush. The following procedures were subsequently performed in each group:

Relined: The glass fiber post (Reforpost Angelus #1, Angelus, Londrina, PR, Brazil) was cleaned with 70% alcohol, followed by the active application of silane (Angelus) for 60 s. A layer of adhesive (Single Bond Universal, 3M ESPE, St. Paul, MN, USA) was applied to the post, the solvent was removed with an air jet, and the adhesive was light-cured for 10 s using a curing light (VALO™ Cordless, Ultradent, South Jordan, UT, USA) at an irradiance of 2,600 mW/cm^2^. The resin composite (Filtek Z350 XT, 3M ESPE) was applied to the apical region of the post. The post was then placed in the canal and light-cured from one side for 5 s using the same curing light and irradiance. Subsequently, the post was removed from the canal for complete light curing and then reinserted to verify adaptation, and the coronal core was built and light-cured for an additional 20 s.

Milled: The root canal was modeled using a post (Pinjet, Angelus) and red acrylic resin (Pattern Bright Kit, Kota Ind., São Paulo, SP, Brazil). The canal model was scanned with CAD software, and the CAM milling machine fabricated the post from a glass fiber block (Fiber CAD Post & Core, Angelus).

Before cementation, 37% phosphoric acid (Ultra Etch, Ultradent) was applied to the post for 30 s, followed by a 30 s rinse. Silane (Angelus) was actively applied to the post for 60 s. The canal was rinsed with distilled water, dried with absorbent paper points, and the root was coated with wax to prevent lateral polymerization during cementation. A dual-cure self-adhesive resin luting agent (RelyX™ U200, 3M ESPE, Seefeld, Germany) was applied directly into the canal with an auto-mix tip. The relined glass fiber post and the CAD/CAM-milled post were inserted, and light curing was performed for 20 s. Finally, the wax surrounding the roots was removed.

### Fracture strength analysis

The roots were embedded in self-curing polystyrene resin (Aerojet, São Paulo, SP, Brazil) with simulated periodontal ligament material (Panasil Initial Contact Light Body Addition Silicone, Ultradent). The roots were marked 2 mm apically from the transverse cut region, and the root portion was coated with a 0.3 mm layer of pink wax to simulate the periodontal ligament. This thickness was confirmed with a digital caliper (Digimess, Shiko Precision Gaging, China). Next, the roots were placed in PVC cylinders with a radiographic film featuring a central perforation compatible with the root's volume. The tooth was positioned and secured to the radiographic film with pink wax, exactly at the 2 mm apical mark from the transverse cut.

Following this, polystyrene resin was prepared according to the manufacturer's instructions and poured into the PVC cylinder. Once the resin had polymerized, the roots were removed from the artificial alveoli and cleaned with water and bicarbonate jets. The silicone-based impression material was prepared following the manufacturer's instructions and inserted into the artificial alveolus, after which the root was reinserted and held under digital pressure until the initially made graphite mark was aligned with the resin cylinder's surface.

The specimens were then subjected to compressive loading in a universal testing machine (DL500, EMIC, Brazil). The specimens were subjected to compressive loading at a 45° angle to the long axis of the tooth at a crosshead speed of 1 mm/min using a universal testing machine equipped with a 200-kgf load cell. The load was applied until a fracture occurred, and the maximum force at failure was recorded in Newtons (N). The fracture load values were recorded and subsequently analyzed statistically, followed by qualitative visual and microscopic assessments.

Qualitatively, the fractures were classified as repairable or catastrophic. The fractures were deemed repairable when they only affected the glass fiber post and/or coronal core or were limited to the cervical region of the root. Fractures involving the middle or apical thirds of the root were classified as catastrophic. The fracture patterns were evaluated independently by two calibrated examiners under optical microscopy at 40× magnification. In cases of disagreement, the specimens were jointly reexamined and a consensus was reached. Given the clearly defined classification criteria, no formal interexaminer agreement analysis was performed.

### Statistical analysis

Fracture strength data were assessed for normality using the Shapiro–Wilk test. As the data showed a normal distribution, fracture strength was compared between the two independent groups using the independent samples *t*-test. The significance level was set at 5% (*α* = 0.05). The statistical analyses were performed using SigmaPlot 14.0 software (Systat Software Inc., San Jose, CA, USA).

## Results

**Figure 1:**
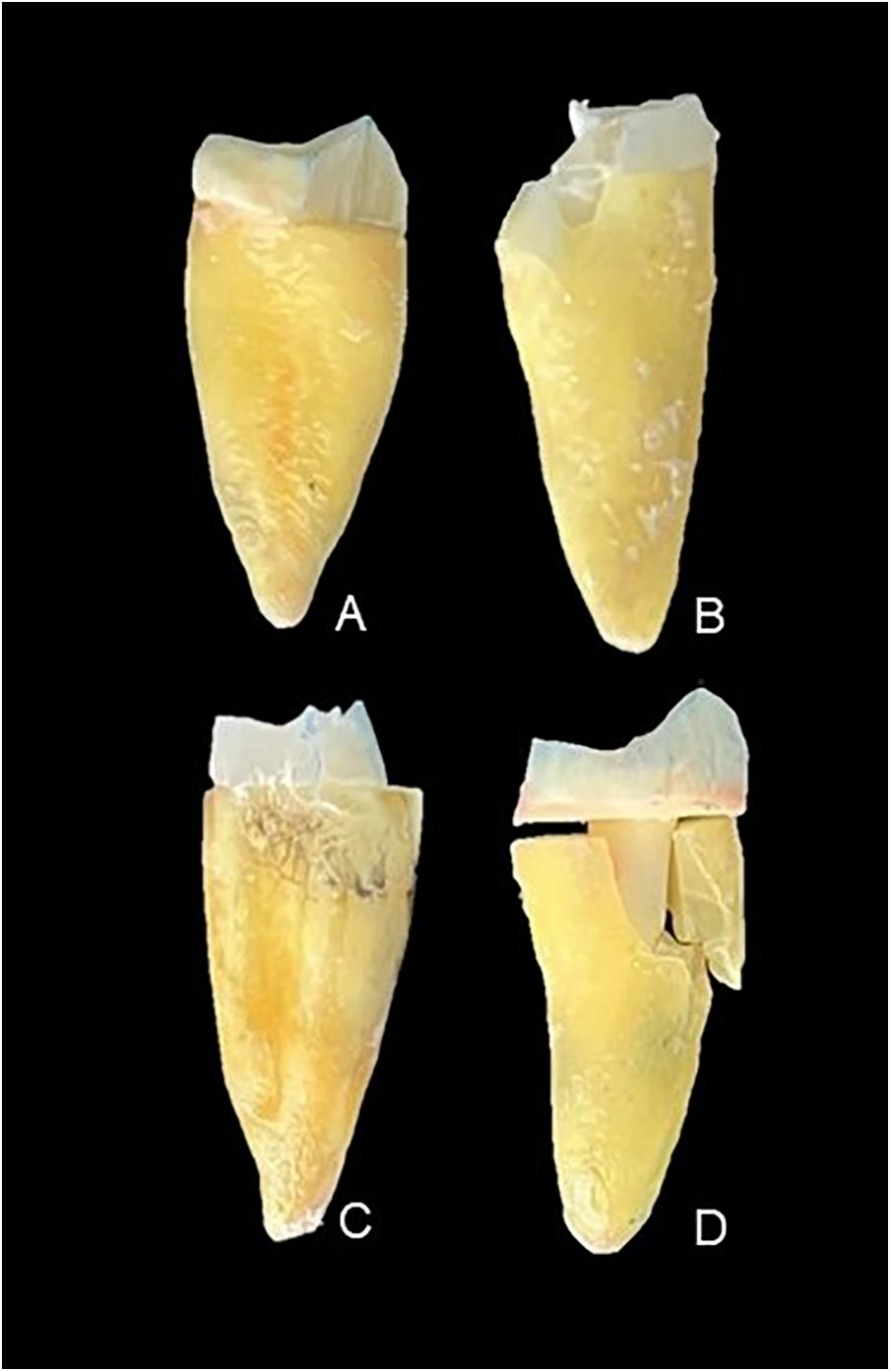
Images of the types of failure observed in the tested groups: **(A)** fracture in the post, **(B)** fracture in the cervical region of the root, **(C)** fracture in the coronal core, and **(D)** fracture in the root at the middle third.

The difference in fracture strength between the groups showed a large effect size (Cohen's *d* = 1.47), with a 95% confidence interval ranging from 0.47 to 2.47, indicating a consistent and clinically relevant difference between the tested post systems. The CAD/CAM-milled glass fiber posts showed higher fracture strength values compared to the relined glass fiber posts (*p* = 0.004) (Table 1). In the relined group (Table 2), root fractures were observed in four specimens, while five showed fractures in the relined glass fiber post. In one specimen, no fracture occurred in either the post or root. Instead, failure occurred solely in the coronal core at the post–resin composite interface. In the milled group, two specimens exhibited root fractures, six showed fractures in the glass fiber post, one displayed a fracture in the coronal core, and one specimen showed a fracture in the cervical region of the root. Figure 1 presents images of the types of failures observed in the specimens.

**Table 1:** Mean and standard deviation (±SD) for the fracture strength (N) of the groups tested.

Group	Fracture strength
Relined	329.5 (111.7) b
Milled	495.0 (113.0) a

**Table 2:** Frequency (%) of the failure modes in the tested groups.

Group	Repairable (%)			Catastrophic (%)
	Post	Cervical	Core	Root
Relined	50	–	10	40
Milled	60	10	10	20

## Discussion

The null hypothesis of this study was rejected. The roots restored with the CAD/CAM-milled glass fiber posts exhibited a higher mean fracture strength than those restored with the relined glass fiber posts, showing a statistically significant difference between these two types of rehabilitation.

When restoring endodontically treated teeth, the success of these rehabilitations depends on selecting materials with a modulus of elasticity similar to that of dental tissues (11), thereby ensuring a better load distribution and protection of the remaining tooth structure (12, 13). The modulus of elasticity of dentin (18 GPa) is closer to that of the glass fiber block used for milling CAD/CAM posts (25 GPa) compared to that of prefabricated glass fiber posts (40 GPa) (11). The higher modulus of elasticity in prefabricated glass fiber posts, combined with that of the resin composite used for relining the post, may reduce fracture resistance in the restoration, increasing the risk of catastrophic fractures. Thermocycling was not included in the experimental protocol, as the study focused on the immediate fracture strength under monotonic loading. Although artificial aging protocols may affect the long-term performance of the adhesive interfaces, all the specimens were tested under identical conditions, which preserves the internal validity of the comparative analysis (3, 10, 14).

Another factor that may have contributed to the higher fracture strength observed in the roots restored with the CAD/CAM-milled posts is the monolithic construction of the post and core, eliminating adhesive interfaces. This single-piece design reduces the risk of failures, as observed in previous studies (11, 14–16). In addition to the monolithic design, the accuracy of digital workflows used for CAD/CAM fabrication has been shown to influence the adaptation and fit of post-and-core restorations. Digitally fabricated posts may exhibit improved precision in post space reproduction, which may contribute to a more favorable stress distribution and biomechanical performance (17).

Recent systematic evidence indicates that CAD-CAM fiber posts may demonstrate higher bond strength and lower catastrophic failure rates compared to prefabricated systems, although clinical data remain limited, underscoring the need for further translational research (18). Furthermore, consistent with our findings, recent systematic reviews have highlighted the influence of material choice and fabrication technique on fracture resistance in endodontically treated teeth restored with fiber-reinforced composites (19).

From a clinical perspective, the fracture strength values observed in the present study exceeded the range of physiological occlusal forces typically reported for anterior teeth, which are generally estimated to vary between approximately 100 and 300 N under normal function (1, 20). Although *in vitro* fracture tests do not directly replicate intraoral conditions, fracture resistance values above these functional loads suggest that both restorative approaches may provide sufficient immediate mechanical support under physiological loading. The higher fracture strength observed for the CAD/CAM-milled glass fiber posts may therefore indicate an increased safety margin against overload conditions, which could be particularly relevant in cases involving parafunctional habits, a compromised remaining tooth structure, or increased functional demands (2, 14, 18). Nevertheless, clinical longevity is influenced by multiple factors beyond immediate fracture resistance, including adhesive durability, fatigue behavior, and biological variables, which reinforces the need for long-term studies to confirm the clinical implications of these findings (8, 20, 21).

A prefabricated post relined with resin composite, with a coronal core also constructed from resin composite, has multiple adhesive interfaces that may increase the risk of failures (7, 9, 20–23). This finding could explain the outcomes observed in the group restored with the prefabricated and relined glass fiber posts, as all the failures in this group, whether affecting the coronal core or the post, occurred precisely at the adhesive interface between the glass fiber post and the resin composite.

Regarding the type of root fracture, it was observed that among the roots restored with CAD/CAM-milled glass fiber posts, two catastrophic fractures occurred that affected the middle third (Table 2; Figure 1). A failure in the middle third is considered irreparable (24). In the samples restored with the prefabricated, relined glass fiber posts, root fractures were observed in four specimens, affecting the middle and apical thirds (Table 2; Figure 1).

These findings are consistent with other studies that have identified catastrophic fractures in roots restored with glass fiber posts (14, 25). All the other specimens that only exhibited post fractures could be restored and were classified as repairable post fractures, a type that preserves root integrity. This outcome can be attributed to effective force dissipation in the root structure (10), which reduces critical stress areas in the dentin walls and helps prevent root fractures (26).

Milled glass fiber posts offer advantages in fracture strength and preservation of root structure due to the lack of an adhesive interface, being fabricated as a single unit, and having a modulus of elasticity closer to dentin. Limitations in this *in vitro* study, such as the use of a single type of resin luting agent, a single root type, and the absence of crowns on the posts and coronal cores, may not fully replicate *in vivo* conditions. Nevertheless, all necessary standardizations were implemented, including root characteristics, storage, endodontic instrumentation, intraradicular preparation length and diameter, and post and core shape, to minimize biases. An important limitation of this *in vitro* study is that the fracture resistance was evaluated under immediate loading conditions, without the application of thermocycling or mechanical fatigue protocols. Aging procedures have been shown to influence adhesive interfaces and may affect the long-term mechanical performance of postretained restorations. Therefore, the results of the present study should be interpreted as representative of the immediate biomechanical behavior of the tested systems. Future studies incorporating artificial aging protocols and cyclic loading are warranted to better simulate clinical conditions and to assess the long-term performance of CAD/CAM-milled and relined glass fiber posts.

## Conclusion

The CAD/CAM-milled glass fiber posts demonstrated superior fracture strength with a lower frequency of catastrophic fractures.

## Data Availability

The original contributions presented in the study are included in the article/Supplementary Material, further inquiries can be directed to the corresponding author.
